# Strategies to approach high performance in Cr^3+^-doped phosphors for high-power NIR-LED light sources

**DOI:** 10.1038/s41377-020-0326-8

**Published:** 2020-05-15

**Authors:** Zhenwei Jia, Chenxu Yuan, Yongfu Liu, Xiao-Jun Wang, Peng Sun, Lei Wang, Haochuan Jiang, Jun Jiang

**Affiliations:** 1 Ningbo Institute of Materials Technology & Engineering, Chinese Academy of Sciences, Ningbo, 315201 China; 2 College of Physics and Optoelectronics, Taiyuan University of Technology, Taiyuan, 030024 China; 3 University of Chinese Academy of Sciences, Beijing, 100049 China; 4 Department of Physics, Georgia Southern University, Statebore, GA 30460 USA

**Keywords:** Atom optics, Lasers, LEDs and light sources, Optical materials and structures

## Abstract

Broadband near-infrared (NIR)-emitting phosphors are key for next-generation smart NIR light sources based on blue LEDs. To achieve excellent NIR phosphors, we propose a strategy of enhancing the crystallinity, modifying the micromorphology, and maintaining the valence state of Cr^3+^ in Ca_3_Sc_2_Si_3_O_12_ garnet (CSSG). By adding fluxes and sintering in a reducing atmosphere, the internal quantum efficiency (IQE) is greatly enhanced to 92.3%. The optimized CSSG:6%Cr^3+^ exhibits excellent thermal stability. At 150 °C, 97.4% of the NIR emission at room temperature can be maintained. The fabricated NIR-LED device emits a high optical power of 109.9 mW at 520 mA. The performances of both the achieved phosphor and the NIR-LED are almost the best results until now. The mechanism for the optimization is investigated. An application of the NIR-LED light source is demonstrated.

## Optics: Visibly powerful near-infrared light-emitting diodes

A near-infrared light-emitting (NIR-LED) diode that emits high-power light could pave the way for the development of next-generation monitoring and detecting devices. Although solid-state NIR-LEDs are used in such devices, their narrow emission band limits their range of applications. Broadband NIR-emitting phosphor-converted LEDs offer the best solution. However, creating NIR phosphors that are sufficiently excited by blue light is challenging. Now, a team of Chinese and American researchers, led by Yongfu Liu from the Chinese Academy of Sciences, has created a NIR-LED that emits light in the 700–900 nm with an output of 109.9 mW at 520 mA after excitation with blue light. The device has the highest recorded power rating to date and could be used in applications from bioimaging and night-vision technologies, to monitoring food and medicines.

## Introduction

NIR spectroscopy has good penetration for organic matter, and it has drawn attention for application in monitoring foods and medicines, bioimaging, and night vision^1–6^. Smart NIR light sources, an emerging field, are proposed to be combined with smart phones to achieve convenient and fast applications^7–9^. In contrast to traditional tungsten filament lamps and halogen lamps, only light-emitting diodes (LEDs) that have a solid state and a small size are suitable for smart NIR devices. However, NIR-LED chips can only give narrow NIR emissions, which limits their applications^10–12^. Broad NIR-emitting phosphor-converted (pc) LEDs, adopting the technology of pc-white LEDs^13–19^, are believed to be the best solution. White LEDs are commonly based on blue-LED chips. Thus, how to achieve broad NIR phosphors that can be efficiently excited by blue light is one of the most important challenges^20^.

Recently, a number of NIR phosphors were realized^21–44^. Among them, Cr^3+^ usually manifests a high efficiency, and the IQE can reach 58–75%^31–37^. The radiant power is 14.7–54.29 mW when driven at 100–130 mA^34–40^. A high radiant power is beneficial for monitoring and detection. Liu et al made great progress in improving the radiant power, which was enhanced from 7–18.2 mW^42,43^ to 65.2 mW^44^ when driven at 350 mA. To achieve high radiance, a high-power LED chip operating at a high current is usually needed. In this situation, the large amount of heat will result in a high temperature on the surface of the chip. Thus, the greatest challenge is how to make NIR phosphors have excellent thermal stability to overcome the thermal quenching effect, in addition to a high QE for NIR phosphors.

We note that Cr^3+^ has a high QE in garnets^34,35^. The silicate garnet Ca_3_Sc_2_Si_3_O_12_ (CSSG) is a promising host for Ce^3+^ due to its excellent thermal stability and high QE^45–47^. Fortunately, Cr^3+^ can be excited by blue light and show broadband NIR emission in CSSG^39^. Unfortunately, the reported luminescence (IQE: 12.8%) and thermal stability were low because the Cr^3+^ luminescence suffered from impurities and oxidation of Cr^4+^ when the material was sintered in air^39^. Based on our previous experience, we propose a strategy to optimize CSSG:Cr^3+^ by enhancing the crystallinity, modifying the micromorphology, and maintaining the valence state of Cr^3+^. By adding fluxes and sintering in a CO reducing atmosphere, the IQE is greatly enhanced to 92.3%. At 150 °C, 97.4% of the NIR emission at room temperature can be maintained, indicating excellent thermal stability. When combined with a high-power 460 nm blue chip, the estimated radiant power of the fabricated pc-LED even reaches 109.9 mW when driven at 520 mA. The properties of both the optimized CSSG:Cr^3+^ and the achieved NIR-LED are almost the best results to date as far as we know. Benefiting from the high radiant power, the pc-NIR-LED device has good application potential in night-vision technology.

In this work, mechanisms for optimization were investigated. The electron–phonon coupling (EPC) mechanism in CSSG that usually determines the Cr^3+^ luminescence is revealed for the first time. Many types of Cr^3+^-doped NIR phosphors have been discovered. We believe that this work provides an effective strategy to optimize and discover more Cr^3+^-doped NIR phosphors using only a simple method. Moreover, this work will advance the development and application of next-generation smart NIR light sources.

## Results

### Optimization of CSSG:Cr^3+^

**Fig. 1:**
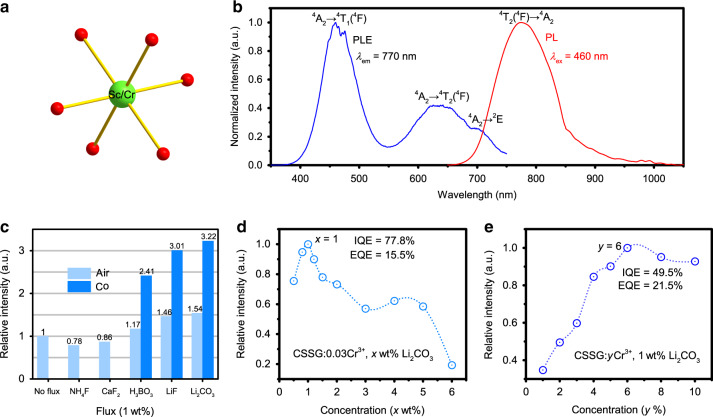
Optimization of the NIR emission. **a** Coordination of (Sc/Cr)O_6_ in CSSG. **b** Photoluminescence (PL) and photoluminescence excitation (PLE) spectra of CSSG:3%Cr^3+^. **c** Relative PL intensities of the samples with and without fluxes sintered in air and a CO atmosphere. **d** PL intensities of CSSG:3%Cr^3+^, *x*wt% Li_2_CO_3_ (*x* = 0.5, 0.8, 1, 1.5, 2, 3, 4, 5, 6). **e** PL intensities of CSSG:y%Cr^3+^, 1 wt% Li_2_CO_3_ (*y* = 1, 2, 3, 4, 5, 6).

CSSG belongs to a cubic crystal system with the space group of Iα3d (Fig. S1). Ca, Sc, and Si are coordinated with 8, 6, and 4 oxygen atoms, respectively^45–47^. Considering the effective ionic radii of Ca^2+^ (1.12 Å), Sc^3+^ (0.745 Å), Si^4+^ (0.26 Å), and Cr^3+^ (*r* = 0.615 Å), it is believed that Cr^3+^ occupies the Sc^3+^ site due to the close radii and same ionic valence^39^. Thus, Cr^3+^ suffers from a weak crystal field (CF) environment in the ScO_6_ octahedron (Fig. 1a). The spin-allowed transitions of ^4^A_2g_ → ^4^T_1g_(^4^F) and ^4^A_2g_ → ^4^T_2g_(^4^F) lead to two excitation bands centered at 460 and 640 nm, respectively (Fig. 1b). The spin-forbidden transition of ^4^A_2g_ → ^2^Eg(^2^G) (R-line) at ~701 nm is also detected. Under 460 nm excitation, CSSG:Cr^3+^ shows a broad NIR emission peaking at ~770 nm with a full-width at half maximum (FWHM) value of ~1750 cm^−1^ (~110 nm), arising from the ^4^T_2g_(^4^F) to ^4^A_2_ transition of Cr^3+^ in the weak CF.

CSSG:Cr^3+^ synthesized in an air atmosphere exhibits a weak NIR emission (Figs. S2–5), which could be attributed to a lower crystallinity and oxidation of Cr^3^^+^ ^39^. For the optimized Cr^3+^ concentration, that is, CSSG:3%Cr^3+^, its IQE and external quantum efficiency (EQE) are quite low, only ~12.8% and 4.8%, respectively (Fig. S6). To enhance the luminescence by improving the crystallinity, fluxes of NH_4_F, CaF_2_, H_3_BO_3_, LiF, and Li_2_CO_3_ were added during the synthesis under the air condition. The NIR emission of the flux-free sample was set as the normalized standard. As Fig. 1c shows, H_3_BO_3_, LiF, and Li_2_CO_3_ enhance the luminescence, while NH_4_F and CaF_2_ decrease the luminescence. Thus, H_3_BO_3_, LiF, and Li_2_CO_3_ were selected, and CSSG:3%Cr^3+^ was sintered in a CO reducing atmosphere to further maintain the state of Cr^3+^. It is noted that the luminescence is greatly enhanced by 2~3 times. Li_2_CO_3_ has the best effect, and the optimal amount is 1 wt% (Fig. 1d). Correspondingly, the IQE and EQE of CSSG:3%Cr^3+^ reach ~77.8% and 15.5%, respectively. Moreover, by adding 1 wt% Li_2_CO_3_ and sintering in CO, the Cr^3+^ concentration is optimized to 6% (Fig. 1e). Then, the achieved EQE of CSSG:6%Cr^3+^ even reaches 21.5%. Here, the QE is the measured result. Due to the limitation of the measurement, up to 850 nm, the results are smaller than the actual values, which will be illustrated later. Therefore, the actual QE is almost the best value among NIR phosphors as far as we know.

### Crystal structures and micromorphology

**Fig. 2:**
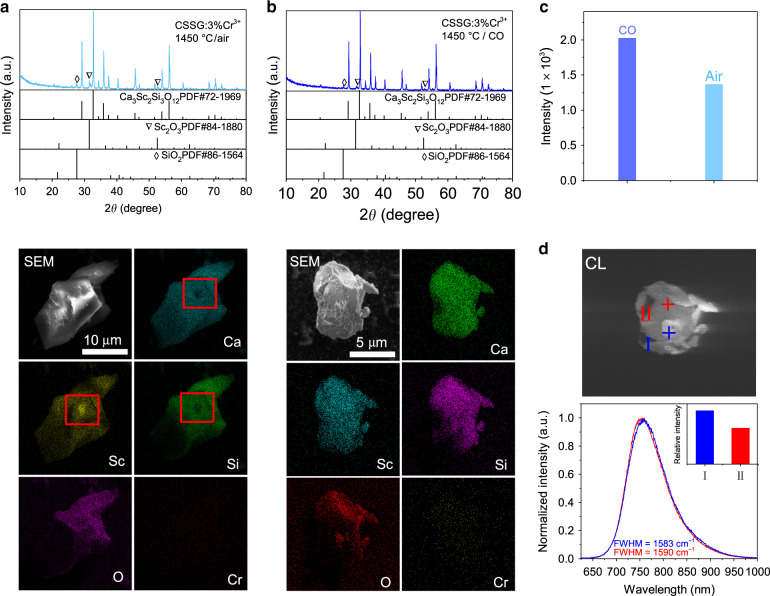
Crystal structures and micromorphology. **a**, **b** XRD patterns, SEM images, and EDS mappings for CSSG:3%Cr^3+^ sintered in air and sintered in CO with 1 wt% Li_2_CO_3_. **c** XRD peak intensities. **d** CL image and normalized CL spectra at points I and II for CSSG:3%Cr^3+^ sintered in CO. The insert shows the CL intensities at points I and II.

To clarify the enhancement of the optimized CSSG:Cr^3+^ compared with the initial phosphor, the crystal structures and micromorphology were studied. For CSSG:3%Cr^3+^ sintered in air (Fig. 2a), the X-ray diffraction (XRD) pattern mainly shows a garnet phase of CSSG (PDF # 72-1969), but a few impurity phases of SiO_2_ and Sc_2_O_3_ are also observed. These impurity phases can also be observed in the SEM-EDS mapping images. Ca, Sc, Si, O, and Cr are inhomogeneously distributed in the square region, in which Sc is rich and Si and Ca are absent.

For CSSG:3%Cr^3+^ sintered in CO with 1 wt% Li_2_CO_3_ (Fig. 2b), the SiO_2_ and Sc_2_O_3_ impurities are quite low in the XRD pattern. The distributions of elements are more homogeneous. In addition, the diffraction peak intensity of the CSSG phase increases (Fig. 2c). These results demonstrate that the enhanced crystallinity of the CSSG phase is one of the reasons for the improvement of the Cr^3+^ NIR emissions.

The CSSG:3%Cr^3+^ phosphor has a particle size of ~10 μm (Fig. 2d). The particle displays a small variation in the cathodoluminescence (CL), as the CL image shows. The bright region (point I) and the dark region (point II) are only different in CL intensity. Their normalized CL spectra are almost the same, having an emission peak at ~760 nm and an FWHM of 1583–1590 cm^−1^ (93–94 nm), similar to the photoluminescence (PL) spectrum in Figs. S2–S5.

### Diagnosis of the valence state of Cr^3+^

**Fig. 3:**
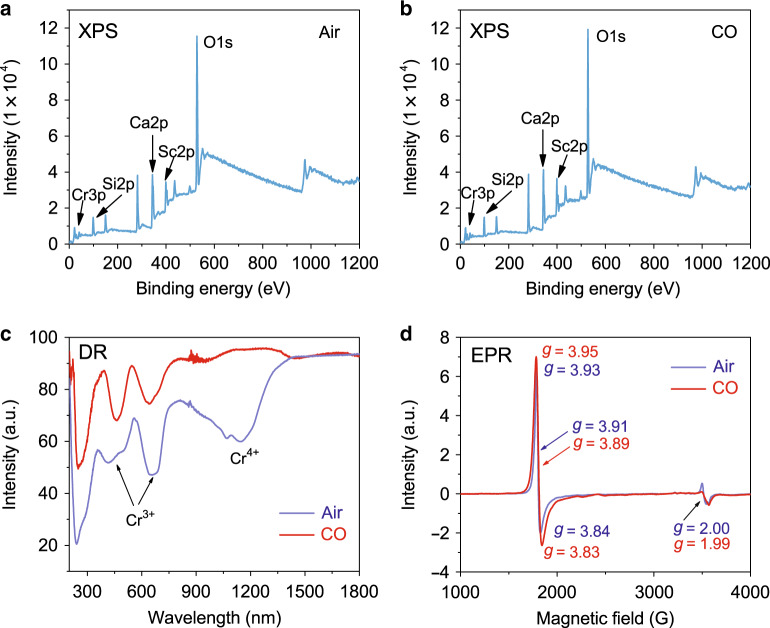
Diagnosis of the valence state of Cr. **a**, **b** XPS, **c** DR, and **d** EPR results of CSSG:3%Cr^3+^ calcined in air and CO.

To diagnose the change in the ionic valence of Cr, X-ray photoelectron spectroscopy (XPS), diffuse reflection (DR), and electron paramagnetic resonance (EPR) results for CSSG:3%Cr^3+^ sintered in air and CO are given in Fig. 3. The binding energies at 99, 344, 399, and 528 eV are from Si-2p, Ca-2p, Sc-2p, and O-1s, respectively. The binding energy at 40 eV for Cr-3p is detected in the two samples^48^. The changes in Cr are not apparent in the XPS spectra. However, in the DR spectra (Fig. 3c), the absorption band of Cr^4+^ (at ~1140 nm) is clearly identified for the sample sintered in air in addition to the absorption band of Cr^3+^ (at ~460 and 640 nm)^34,35^. This means that some Cr^3+^ ions are oxidized into Cr^4+^ even though Cr_2_O_3_ is used as the raw material. For the sample sintered in CO, the Cr^4+^ absorption band almost disappears, and only the Cr^3+^ absorption band is observed. This means that Cr^3+^ is well maintained in the CO reducing atmosphere.

In the 3d^3^ electronic configuration of Cr^3+^, three electrons occupy the d orbitals and give rise to a total spin of S = 3/2. ^4^A_2g_(F) is the ground state for Cr^3+^. In an octahedral crystal field, the ^4^F state splits into a singlet orbital ^4^A_2g_ and two orbitals ^4^T_1g_ and ^4^T_2g_, thus causing EPR signals^49^. As Fig. 3d shows, the sharp peaks at *g* = 3.8–3.9 belong to the isolated Cr^3+^ ion, and the peaks at approximately *g* = 2 represent the first neighbor Cr^3+^ –Cr^3+^ pair^42–44,49^. The EPR intensity for the sample sintered in CO is stronger than that for the sample sintered in air, demonstrating an increased ratio of Cr^3+^. Combining the DR and EPR results, it is claimed that Cr^3+^ can be maintained and increased in the reducing atmosphere, which is another reason for the improvement of the Cr^3+^ NIR luminescence.

### Temperature-dependent NIR emissions

**Fig. 4:**
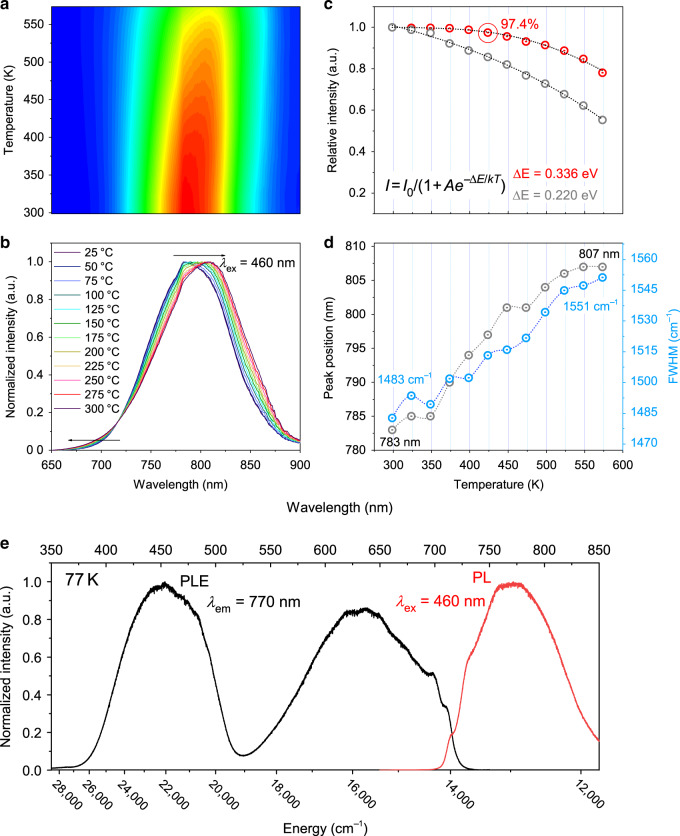
Luminescence dependence on temperature for CSSG:6%Cr^3+^. **a** PL and **b** normalized PL spectra. **c** Integrated PL intensities for the sample sintered in air and the optimized sample. The dotted line is the fitting result for the Arrhenius equation. **d** Peak positions and FWHM values. The temperature ranges from 25 to 300 °C. **e** PL and PLE spectra at 77 K.

Figure 4a–c shows the temperature-dependent luminescence of CSSG:6%Cr^3+^. The integrated PL intensity at 25 °C is set as the normalized standard, and 97.4% can still be maintained at 150 °C for the phosphor sintered in CO, whereas 85.6% can be maintained for the phosphor sintered in air. The temperature dependence of the emission intensity can be well fitted by the Arrhenius formula, and the activation energy is calculated to be ∆*E* = 0.336 eV for the optimized sample, compared with ∆*E* = 0.220 eV for the initial sample (Fig. 4b). When the temperature increases from 25 to 300 °C, the peak position redshifts from ~783 to ~807 nm (Fig. 4d), attributed to the decreased CF caused by lattice expansion. The FWHM increases from 1483 to 1551 cm^−1^ (92.3 to 100.2 nm), attributed to the strengthened EPC effect that will be discussed in the following.

Figure 4e shows the PLE and PL spectra of CSSG:6%Cr^3+^ at 77 K with a step size of 0.05 nm. The transitions from ^4^A_2g_ to ^4^T_2g_(^4^F) and ^4^T_1g_(^4^F) are centered at 15,670 cm^−1^ (~636 nm) and 22,050 cm^−1^ (~453 nm), respectively. The R-line is only detected in the PLE spectrum at 698.3 nm (~14,320 cm^−1^). The peak at ~713 nm (~14,030 cm^−1^) is observed in both the PL and PLE spectra, which is assigned to the zero-phonon line (ZPL) for the ^4^T_2_(^4^F) and ^4^A_2g_ transition (Fig. S7). The energy gap between ^2^E_g_(^2^G) and the ^4^T_2g_(^4^F) ZPL was evaluated to be ~290 cm^−1^, indicating a strong spin–orbit coupling (SOC) of the ^2^E_g_(^2^G) and ^4^T_2g_(^4^F) states^50^. Thus, the fluorescent decay curve shows a biexponential model with a lifetime of ~190 µs (Fig. S8–S9).

The PL spectrum has a maximum peak at ~12,980 cm^−1^ (~770 nm) with a FWHM of 1560 cm^−1^ (~93 nm). The corresponding Stokes shift is ~2700 cm^−1^. The peak at 730 nm (~13,700 cm^−1^) is the phonon satellite of the ZPL. The energy difference between the ZPL and its phonon satellite is determined to be ~330 cm^−1^, which corresponds to one of the vibrational modes (*ħw*) that couple with the ^4^T_2g_(^4^F) → ^4^A_2g_ transition (Fig. S7).

A Stokes shift has a relationship of (2*S* + 1)*ħw*, where *S* is the Huang–Rhys parameter; thus, *S* is determined to be between 3 and 4, which is smaller than the value (~6) for LSGG:Cr^3+^ ^31^. Both the broadband emissive characteristic and the large Stokes shift indicate a stronger EPC for the ^4^T_2g_(^4^F) → ^4^A_2g_ transition in CSSG:Cr^3+^. A stronger EPC leads to a larger *S* value^28–31^. At a low temperature (77 K), the EPC effect is weak. Thus, the FWHM value at 77 K decreases by ~190 cm^−1^ compared with the FWHM of 1750 cm^−1^ (~110 nm) at RT.

It is worth noting that the ratio in the range of 650–725 nm increases and the emission shows a blueshift with increasing temperature. The small energy gap (~ 290 cm^−1^) between ^2^E_g_ and the ^4^T_2g_ ZPL leads to mixing of the ^2^E_g_(^2^G) and ^4^T_2g_(^4^F) states. When the temperature increases, electronic transfer from the ^4^T_2g_ state to the ^2^E_g_ state is strengthened with the assistance of EPC. Thus, the radiative transitions from the ^2^E_g_(^2^G) state increase, and blueshifts are observed.

### Performance of the fabricated NIR-LED device

**Fig. 5:**
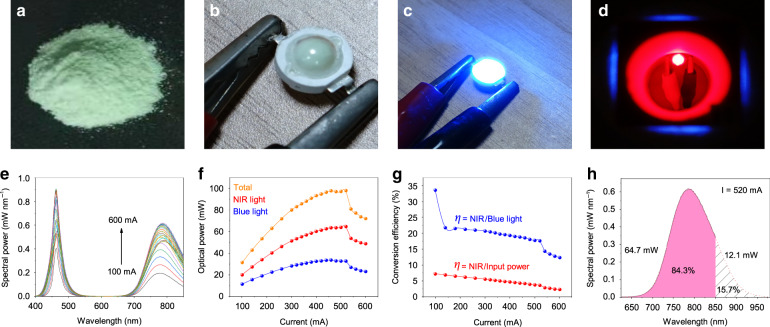
Performance of the fabricated high-power NIR-LED device. **a** Body color of the CSSG:6%Cr^3+^ phosphor under sunlight. **b** NIR-LED device fabricated using the optimized phosphor and a 460 nm blue LED. **c** Working state of the NIR-LED taken without a filter and **d** with a longpass filter at 650 nm. **e** EL spectra, **f** output optical powers, and **g** conversion efficiencies of the NIR-LED depending on the driving current. **h** EL spectrum fitted by the Gaussian formula.

The CSSG:6%Cr^3+^ phosphor shows a green color (Fig. 5a). Based on the optimized phosphor and a high-power blue chip, an NIR-LED device was fabricated and is displayed in Fig. 5b–d. The electroluminescence (EL) spectra, optical powers, and conversion efficiencies of the device depending on the driving current (*I*) are given in Fig. 5e–h. The strong emission peak at ~460 nm comes from the blue chip. The broad NIR emission band comes from CSSG:Cr^3+^. The optical powers of both the total radiance and NIR light increase with increasing current until reaching maxima of 97.8 and 64.7 mW, respectively, at 520 mA.

The conversion efficiency from the emitted blue light to NIR light (η_NIR/blue light_) drops from 33.5 to 12.3% when the driving current increases from 100 to 600 mA. Correspondingly, the conversion efficiency from the input electronic power to NIR emissions (η_NIR/input_) decreases from 7.2 to 2.3%. η_NIR/input_ is much lower than η_NIR/blue light_. The lower photoelectric conversion efficiency (η_blue light/input_) from the input electronic power to blue light should be responsible for this phenomenon because the η_blue light/input_ of the used blue chip, ranging from 31.9 to 19.4% at 100–600 mA, is not very high (Fig. S9). If the used blue chip is efficient, then η_NIR/input_ can be greatly enhanced further.

On the other hand, only 84.3% of the NIR emission can be detected due to the limitation of the measurement range, up to 850 nm (Fig. 5h). If the 15.7% unmeasured part is taken into account, then the actual NIR optical power should be 76.8 mW at 520 mA. Thus, the total optical power even reaches 109.9 mW, which is much higher than the performances reported until now^44^.

The QEs discussed above are the measured results. The QE and radiant power were measured by using the same spectrometer. If the unmeasured part is also taken into account, then the IQE and EQE can actually reach 92.3% and 25.5%, respectively, which are almost the best results as far as we know up to now.

### Applications in night vision

**Fig. 6:**
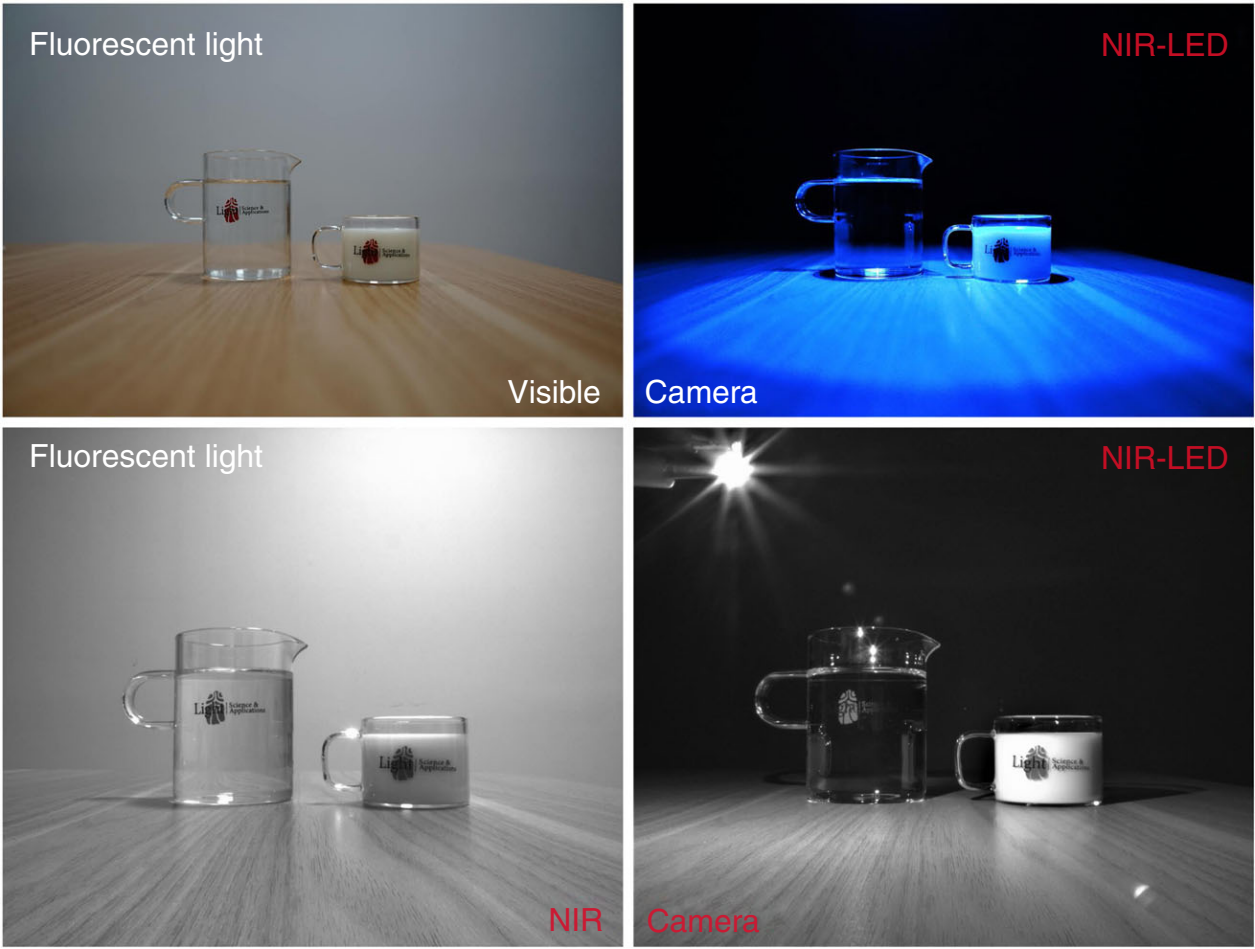
Applications for night vision. Visible images and NIR images of water and milk illuminated by fluorescent light and the fabricated NIR-LED light.

Figure 6 shows the application of the NIR-LED for night vision. Visible images taken by a visible camera are colorful when water, milk, and cups are illuminated by either fluorescent light or NIR light. The logo is clear under fluorescent light. However, only black-and-white images are captured by an NIR camera. When the NIR-LED is off, nothing can be captured. When the NIR-LED is on, the logo is much clearer when it is taken by the NIR camera than when it is taken by the visible camera, especially the logo on the surface of the glass filled with transparent water. These results indicate that the achieved CSSG:Cr^3+^ phosphor enables the NIR-LED to have good application in night-vision technology. Potential applications in monitoring foods and medicines are also expected for such NIR phosphors and NIR-LED light sources.

## Discussion

In conclusion, CSSG:Cr^3+^ exhibits broad NIR emission from 700 to 900 nm under blue light excitation. The previously reported CSSG:Cr^3+^ synthesized in air has low IQE (12.8%) and EQE (4.8%), limiting its performance in pc-NIR-LED devices. By adding fluxes and synthesizing in a CO reducing atmosphere, the IQE and EQE are greatly improved to 77.8% and 21.5%, respectively. If the unmeasured part is taken into account, then the actual IQE and EQE should reach 92.3% and 25.5%, respectively, which is almost the best result among the NIR phosphors developed until now. Investigation of the crystal structures and micromorphology demonstrated that the improvement arises from the modification of the crystallinity and the maintenance of Cr^3+^. The achieved CSSG:6%Cr^3+^ exhibits excellent thermal stability, and 97.4% of the emission intensity at room temperature can still be maintained at 150 °C. Thus, when it was used in a high-power blue chip, the fabricated NIR-LED showed a high optical power of nearly 110 mW at 520 mA, which is almost the best performance among NIR-LED light sources.

Among the reported NIR phosphors, Cr^3+^ is an important activator, and its luminescence is determined by both the selected host and the synthesis technology. We believe that this work provides an effective strategy to optimize NIR phosphors using only a simple but the best method. Thus, it will inspire more researchers to achieve much better performance of known NIR phosphors and advance development of next-generation smart NIR-LED light sources.

## Materials and methods

### Synthesis

Samples with the nominal composition of Ca_3_Sc_2-x_Si_3_O_12_:*y*Cr^3+^, *x*wt% flux were synthesized by a high-temperature solid-state reaction. The starting materials of CaCO_3_ (99.9%), Sc_2_O_3_ (99.9%), SiO_2_ (AR), and Cr_2_O_3_ (99.95%) and fluxes of NH_4_F, CaF_2_, H_3_BO_3_, LiF, and Li_2_CO_3_ were weighed according to the nominal composition and then ground in an agate mortar for 30 min. After that, the powders were sintered at 1450 °C for 3 h in air and a CO atmosphere.

### Fabrication of pc-NIR-LEDs

NIR-LEDs were fabricated using the optimized NIR phosphor CSSG:6%Cr^3+^ and high-power blue-LED chips (460 nm). The phosphors were thoroughly mixed with epoxy resin and then coated on the chips.

### Characterization

XRD patterns were measured by a Bruker D8 X-ray diffractometer with Cu Kα radiation (*λ* = 1.54056 Å) at 40 kV and 40 mA. DR spectra were measured by a LAMBDA 950. PL and PLE spectra at RT-300 °C were characterized by a Hitachi F-4600. PL and PLE spectra at 77 K were measured by a Horiba FL-311 by dipping the sample in liquid nitrogen. EPR data were recorded on a Bruker E500 with the X-band frequencies (≈9.845 GHz) and a microwave power of 0.63 mW. XPS was performed on a Kratos Axis Ultra DLD. IQEs and EQEs were recorded by an Otsuka Photal Electronics QE-2100. A field-emission scanning electron microscope (FE-SEM, Hitachi S-4800) equipped with an energy dispersive X-ray spectroscopy (EDS) system and a CL system (MonoCL4, Gatan) was used to measure the morphology. EL spectra and performances of fabricated pc-NIR-LED devices were measured by an integrating sphere (Labsphere), and data were collected by a multichannel photodetector (MCPD-9800, Otsuka Photal Electronics). Visible images and NIR images were taken by a visible camera (SONY ILCE-7M2K) and an NIR camera (Work Power UC-500M), respectively.

## Supplementary information

Supplementary Information
