# Supplementary material for: Strategies to approach high performance in Cr3+-doped phosphors for high-power NIR-LED light sources
Source: Light Sci Appl. 2020 May 15;9:86. doi: 10.1038/s41377-020-0326-8 (PMC7229223; doi:10.1038/s41377-020-0326-8)
Supplement: Supplementary file 1 — Supplementary Information [file 41377_2020_326_MOESM1_ESM.docx]

**Supplementary Information**

**Strategies to approach high performance in Cr^3+^-doped phosphors for high-power NIR-LED light sources**

Zhenwei Jia^1, 2, #^, Chenxu Yuan^1, 3, #^, Yongfu Liu^1, *^, Xiao-Jun Wang^4^, Peng Sun^1^, Lei Wang^2, *^, Haochuan Jiang^1^ and Jun Jiang^1^

^1^ *Ningbo Institute of Materials Technology & Engineering, Chinese Academy of Sciences, Ningbo 315201, PR China*

^2^ *College of Physics and Optoelectronics, Taiyuan University of Technology, Taiyuan 030024, PR China*

^3^ *University of Chinese Academy of Sciences, Beijing 100049, P. R. China*

^4^ *Department of Physics, Georgia Southern University, Statebore, GA 30460, USA*

^#^ These authors contributed equally.

^*^Correspondence: Yongfu Liu ([liuyongfu@nimte.ac.cn](mailto:liuyongfu@nimte.ac.cn)) or Lei Wang ([wanglei_keke@163.com](mailto:wanglei_keke@163.com))

**Fig. S1** Crystal structure of CSSG viewed along the *a* axis and coordination environments of Ca^2+^, Sc^3+^, and Si^4+^.





**Fig. S2 PLE and PL spectra. a** Photoluminescence excitation (PLE) and **b** photoluminescence (PL) spectra of CSSG:3%Cr^3+^ without and with 1wt% fluxes of CaF_2_, H_3_BO_3_, NH_4_F, LiF, and Li_2_CO_3_ sintered in air. The strong excitation band at ~210 nm should belong to the O^2−^ - Cr^3+^ charge transfer band. The PLE band at 460 nm from transitions of ^4^A_2g_ → ^4^T_1g_(^4^F). The PL peak positions (~ 783 nm) and the band widths (103-106 nm) almost do not change.

**Fig. S3 PL spectra.** PL spectra of CSSG:3%Cr^3+^ without flux sintered in air and with 1wt% fluxes of H_3_BO_3_, LiF, and Li_2_CO_3_ sintered in the CO reducing atmosphere. The PL peak positions (~ 783 nm) and the band widths (~ 103 nm) almost do not change.





**Fig. S4 PLE and PL spectra. a** PLE and **b** PL spectra of CSSG:3%Cr^3+^, *x*wt%Li_2_CO_3_ (x = 0.5-6) sintered in the CO reducing atmosphere. The PL peak positions (~ 783 nm) and the band widths (~ 103 nm) almost do not change.

.





**Fig. S5 PLE and PL spectra. a** PLE and **b** PL spectra of CSSG:*x*Cr^3+^, 1wt% Li_2_CO_3_ (*x* = 0.01 - 0.1). The PL peak positions (~ 783 nm) and the band widths (~ 103 nm) almost do not change.

**Fig. S6** Internal quantum efficiency (IQE) and external quantum efficiency (EQE) of CSSG:3%Cr^3+^, sintered in air, CSSG:3%Cr^3+^,1wt% Li_2_CO_3_ and CSSG:6%Cr^3+^,1wt% Li_2_CO_3_ sintered in the CO atmosphere.


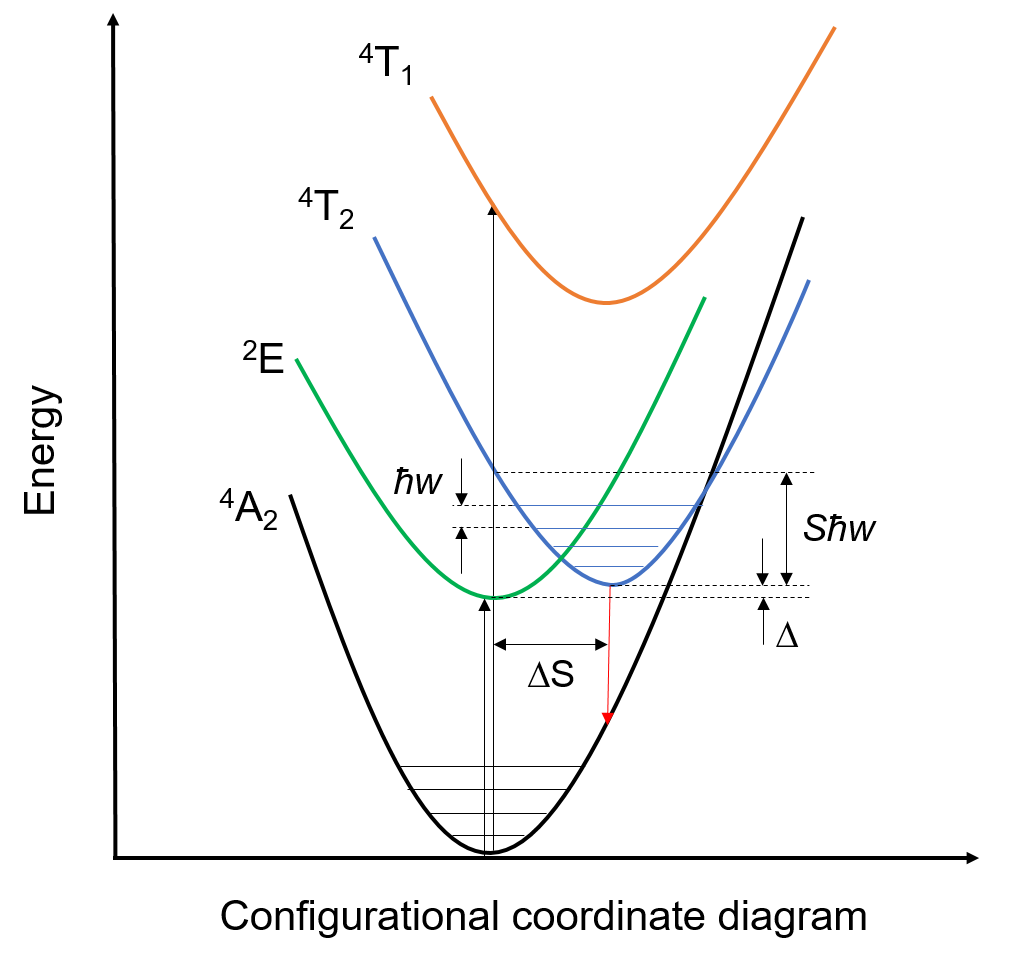


**Fig. S7** Configurational coordinate diagram of CSSG:Cr^3+^. The ħw represents phonon energy, *S* represents Huang-Rhys factor, ∆S is the Stokes Shift.





**Fig. S8** Tanabe–Sugano energy level diagram for Cr^3+^ (3d^3^) in an octahedral crystal field.

Using formulas proposed by Henry, Tanabe, and Sugano:

$E\left( {{}^{4}T}_{2} \right)=10Dq$ (1)

$\frac{Dq}{B}=\frac{15\times\left( \frac{\Delta E}{Dq}-8 \right)}{\left( \frac{\Delta E}{Dq} \right)^{2}-10\times\frac{\Delta E}{Dq}}$ (2)

$\Delta E=E\left( {}^{4}{T_{1}} \right)-E\left( {}^{4}{T_{2}} \right)$ (3)

$E\left( {}^{2}E \right)=3.05C+7.9B-\frac{1.8B^{2}}{Dq}$ (4)

where B and C are the Racah electron repulsion parameters, E(^4^T_1_) and E(^4^T_2_) correspond to the energy level position of ^4^T_1__g_(^4^F) and ^4^T_2__g_(^4^F), and ∆E represents the energy difference between the two levels. Dq, B, and C are estimated to be about 1565, and 571, and 3330 cm^-1^, respectively. The crystal field strength Dq/B is ~ 2.74, which is quite different from Ref. 39 (Dq/B ~ 2.25). A small difference in determining the ^4^T_1__g_(^4^F) and ^4^T_2g_(^4^F) energy positions will lead to a great difference in Dq/B.

**Fig. S9** Fluorescent decay curve of CSSG:6%Cr^3+^, 1wt% Li_2_CO_3_ monitored at 780 nm under 460 nm excitation. The black line represents the fitting curve by a biexponential function. The average lifetime is 187.9 µs. These phenomena could be attributed to the small energy gap between the ZPL of ^4^T_2g_(^4^F) and ^2^E_g_(^2^G) energy level, which leads to a strong mixing of the ^2^E_g_ and ^4^T_2g_(^4^F) states by spin-orbit coupling.





**Fig. S10** Output optical power and photoelectric efficiency of the used 460 nm blue chip.

**

**

**Fig. S11** Luminous efficacy of the NIR LED dependent on driven currents. The luminous efficacy of the NIR LED decreases from 3.3 to 1 lm W^-1^ when current increases from 100 to 600 mA.
